# Whole-Genome Resequencing Reveals Genetic Diversity and Wool Trait-Related Genes in Liangshan Semi-Fine-Wool Sheep

**DOI:** 10.3390/ani14030444

**Published:** 2024-01-29

**Authors:** Xueliang Sun, Jiazhong Guo, Ran Li, Huanhuan Zhang, Yifei Zhang, George E. Liu, Quzhe Emu, Hongping Zhang

**Affiliations:** 1 Key Laboratory of Livestock and Poultry Multi-Omics, Ministry of Agriculture and Rural Affairs, College of Animal Science and Technology, Sichuan Agricultural University, Chengdu 611130, China; sunxueliang97@163.com (X.S.); jiazhong.guo@sicau.edu.cn (J.G.);; 2 Farm Animal Genetic Resources Exploration and Innovation Key Laboratory of Sichuan Province, Sichuan Agricultural University, Chengdu 611130, China; 3 Key Laboratory of Animal Genetics, Breeding and Reproduction of Shaanxi Province, College of Animal Science and Technology, Northwest A&F University, Yangling 712100, China; 4 Animal Genomics and Improvement Laboratory, Beltsville Agricultural Research Center, Agricultural Research Service, USDA, Beltsville, MD 20705, USA; 5 Animal Genetics and Breeding Key Laboratory of Sichuan Province, Sichuan Animal Science Academy, No. 7, Niusha Road, Chengdu 610066, China

**Keywords:** Chinese sheep, wool, whole-genome sequencing, genetic diversity, selection signature, runs of homozygosity, inbreeding coefficients

## Abstract

**Simple Summary:**

The Liangshan semi-fine-wool sheep is a composite breed, developed in Southwest China by crossing local coarse-wool sheep (Liangshan native sheep), fine-wool sheep (e.g., Xinjiang Merino sheep), and semi-fine-wool sheep (e.g., Border Leicester and Romney sheep). This breed not only has soft wool with a spinning count from 48 to 50s but also exhibits excellent adaptability to the local cold and humid environment. Nevertheless, recent declines in wool market demand and the lack of continuous genetic improvement initiatives have resulted in a reduction in its population size. In this study, we systematically investigated the genetic composition of this breed through comparative genomic analyses. Compared to Chinese sheep populations (e.g., Yunnan sheep), our findings unveiled a shorter genetic distance between Liangshan semi-fine-wool sheep and non-Chinese breeds like Border Leicester and Romney sheep. Additionally, we identified many plausible candidate genes and signaling pathways associated with wool traits specific to Liangshan semi-fine-wool sheep. These results provide important references and genomic resources that can be harnessed for future genetic breeding improvements for Liangshan semi-fine-wool sheep.

**Abstract:**

Understanding the genetic makeup of local sheep breeds is essential for their scientific conservation and sustainable utilization. The Liangshan semi-fine-wool sheep (LSS), a Chinese semi-fine-wool breed renowned for its soft wool, was analyzed using whole-genome sequencing data including 35 LSS, 84 sheep from other domestic breeds, and 20 Asiatic mouflons. We investigated the genetic composition of LSS by conducting analyses of the population structure, runs of homozygosity, genomic inbreeding coefficients, and selection signature. Our findings indicated that LSS shares greater genetic similarity with Border Leicester and Romney sheep than with Tibetan (TIB), Yunnan (YNS), and Chinese Merino sheep. Genomic analysis indicated low to moderate inbreeding coefficients, ranging from 0.014 to 0.154. In identifying selection signals across the LSS genome, we pinpointed 195 candidate regions housing 74 annotated genes (e.g., *IRF2BP2*, *BVES*, and *ALOX5*). We also found the overlaps between the candidate regions and several known quantitative trait loci related to wool traits, such as the wool staple length and wool fiber diameter. A selective sweep region, marked by the highest value of cross-population extended haplotype homozygosity, encompassed *IRF2BP2*—an influential candidate gene affecting fleece fiber traits. Furthermore, notable differences in genotype frequency at a mutation site (c.1051 + 46T > C, Chr25: 6,784,190 bp) within *IRF2BP2* were observed between LSS and TIB and YNS sheep (Fisher’s exact test, *p* < 2.2 × 10^−16^). Taken together, these findings offer insights crucial for the conservation and breeding enhancement of LSS.

## 1. Introduction

Sheep (*Ovis aries*) were among the earliest animals to be domesticated, occurring ap-proximately 10,000 to 12,000 years ago in the Fertile Crescent [1,2]. Since domestication, sheep have served as sources of mutton, wool, skin, and milk for humans. In particular, wool traits were one of the driving factors during the sheep expansion history [3]. Due to its excellent insulation and appearance, wool has been a significant commodity for human beings, particularly in ancient societies [4]. As a result of long-term artificial directional selection and environmental adaptability, a variety of wool breeds with distinct fiber diameters have been formed [5]. For example, Chinese Merino sheep (CNM) are well known for fine wool that is highly sought in global luxury fabric manufacture [6]. Meanwhile, semi-fine-wool, exemplified by breeds such as Border Leicester (LST) and Romney (RMN), finds common use in textile applications due to its wool fineness [7,8]. By contrast, the wool of many Chinese indigenous breeds including Tibetan sheep (TIB) is coarse and used in clothing manufacturing, providing a balance of decent thermal insulation and cost-effectiveness [9].

Wool traits (such as fiber diameter and staple length) are economically important traits influenced by many genetic factors [10,11], and therefore there are long-term interests to elucidate the molecular mechanisms and identify the genetic loci underlying these traits. Wool hair follicles experience cycles of growth (anagen), apoptosis-mediated regression (catagen), and relative quiescence (telogen) [12,13]. Within the anagen bulb of the hair follicle, there are matrix keratinocytes and the hair follicle pigmentary unit. The activated matrix keratinocytes, having migrated from the bulge to occupy the matrix region, are highly proliferative cells. The quantity of these cells plays a pivotal role in determining hair bulb size and hair shaft diameter [14]. At the cellular level, the development of hair follicles in sheep constitutes a complex process, which arises from interactions between epidermal keratinocytes committed to hair-specific differentiation and clusters of dermal fibroblasts that form follicular papillae [15]. Previous studies have reported various signaling pathways—WNT, BMP, and FGF signaling—associated with follicle morphogenesis and regeneration [16,17]. At the individual level, a genomic region including *IRF2BP2* was recently determined as a major locus in Altay, Baidarka, and Diqing wool sheep breeds [18,19]. Several candidate genes, including *KRTCAP3*, *KAP*, and *TPTE2*, have been associated with wool traits [20,21]. However, it is important to examine whether these loci hold true for other wool sheep breeds. 

Liangshan semi-fine-wool sheep (LSS) is a composite breed developed in Southwest China, known for its high-quality wool highly favored by consumers and its adaptability to local cold and humid environments. However, the genetic basis of wool-related traits in this breed remains elusive. Throughout the breeding history of LSS, several sheep breeds have been introduced for genetic improvements of wool traits, including CNM sheep recognized for its fine wool and adaptability, and LST sheep originating from the Border region in the United Kingdom, renowned for its exceptional wool yield [22]. According to oral communications, Hu, Australian White, or other sheep breeds have also been introduced in recent years to enhance ewe fertility or mutton production traits in LSS. Furthermore, a recent decrease in wool market demand has led to a decline in the population size of LSS. Consequently, the precise genetic relationship between contemporary LSS populations and other sheep breeds remains uncertain. Therefore, assessing the genomic diversity of LSS becomes essential, as it could significantly contribute to the conservation and utilization of this breed.

A comprehensive understanding of genetic diversity based on high-density SNP data will enable a more accurate assessment of genetic adaptations of domestic sheep to local environments, as well as their future evolutionary potential [23]. In this study, we generated whole genome sequencing (WGS) data for 35 individuals in LSS from Butuo County, Sichuan Province, China. Combining these data with publicly available data from 20 TIB, 20 Yunnan sheep (YNS), 20 CNM, 20 LST, 4 RMN, and 20 Asiatic mouflons (AMU), our primary objective is to investigate the population structure, genetic diversity, runs of homozygosity (ROH), genome-wide inbreeding coefficients, and selection signatures in LSS.

## 2. Materials and Methods

### 2.1. Samples, DNA Extraction, and Whole-Genome Sequencing Data

Thirty LSS sheep were randomly selected and their blood samples were collected from a core sheep breeding farm located in Butuo County, within the Liangshan Yi Autonomous Prefecture, Sichuan Province, China, in 2022. We also used ear tissue samples of five additional LSS animals that were collected from the same farm in 2005. Since the pedigrees were unavailable, we were not sure whether the LSS samples were genetically related or not.

Genomic DNA from a total of 35 animals was extracted through the standard procedure from either whole blood or ear tissue samples, with the Genomic DNA Kit (Tiangen Biotech, Beijing, China). Paired-end sequencing libraries (2 × 150 bp) were constructed for each sheep genome, with an average insert size of approximately 400 bp. The genome sequencing was then conducted on the BGI-T7 platform at Personal Biotechnology in Shanghai, China.

To explore the genetic relationship between LSS and other domestic sheep populations as well as wild sheep, we downloaded WGS data for 20 TIB (NCBI accession number: PRJNA835294), 20 YNS (NCBI accession number: PRJNA802268), 20 CNM (NCBI accession number: PRJNA624020), 20 LST (NCBI accession number: PRJNA325682), 4 RMN (NCBI accession number: PRJNA645671, PRJNA160933, and PRJNA784357), and 20 AMU sheep (NCBI accession number: PRJNA624020 and PRJEB3139) from publicly available databases.

### 2.2. Alignment of Short-Reads and Variant Calling

Raw reads were quality-screened and trimmed for adapters using fastp (v0.36) [24], and processed to obtain high-quality reads. The fastp parameters were set as follows: ‘-q 20 -u 20 -n 5 -l 80 -w 8‘. The high-quality 150-bp paired-end reads were aligned to the sheep genome assembly ARS-UI_Ramb_v2.0 [25] (https://www.ncbi.nlm.nih.gov/datasets/genome/GCF_016772045.1/, accessed on 27 February 2023) along with an additional Y chromosome using the Burrows–Wheeler aligner (BWA mem). Subsequently, the removal of duplicated reads and the recalibration of base quality scores were executed using the Genome Analysis Toolkit software (GATK, v 4.0.5.2). The short genomic variants were called in each sheep sample using the ‘HaplotypeCaller’ parameter in GATK. Then, all individual sheep files were merged into a single gVCF file with the ‘CombineGVCFs’ module in the GATK software, followed by VariantFiltration using the settings ‘QUAL < 100.0 || MQ < 40.0 || FS > 60.0 || QD < 2.0 || SOR > 3.0 || ReadPosRankSum < −8.0 || MQRankSum < −12.5‘. Ultimately, we used VCFtools (v0.1.16) [26] to filter out variants having a minor allele frequency (MAF) less than 0.05 and over 10% missing genotype data. Also, SNP variant annotation and effect prediction were conducted using the SnpEff software (v4.3) [27].

### 2.3. Phylogenetic, Principal Component, and Population Structure Analysis

Based on previous studies [28,29], we used independent markers to perform phylogenetic, principal component (PCA), and population structure analyses to avoid the bias resulting from closely linked SNPs. Utilizing the common empirical values in previous studies [30,31,32], we pruned the SNPs in the high levels of linkage disequilibrium (LD) using PLINK (v1.9) [33] with the parameter ‘--indep-pairwise 50 5 0.2’. The neighbor-joining phylogenetic tree was constructed using MEGAX software (v11.0.13) [34] after calculating the identity-by-state matrix among all 139 sheep samples with PLINK [33]. Subsequently, we utilized iTOL (https://itol.embl.de/, accessed on October 18, 2023) to visualize the phylogenetic tree. PCA was conducted using GCTA (v1.92) [35] (--pca 20); the first two components were utilized to differentiate population structure. The results were visualized using the R package ggplot2 [36]. We performed population structure analysis in the sampled sheep using ADMIXTURE (v1.3) [37] (K = 2 to K = 7). We computed the outgroup f3-statistics (X, Y; AMU) using the qp3Pop parameter of ADMIXTOOLS (v7.0.2) software [38]. The genome-wide fixation index (*F*_ST_) was also calculated with 10-kb non-overlapping windows using the ‘--weir-fst-pop’ parameter in VCFtools (v0.1.16) [26].

### 2.4. Genetic Diversity and Genome-Wide Detection of ROHs

The genome-wide nucleotide diversity (π) within the LSS breed was calculated using VCFtools (v0.1.16) software [26] with 10-kb non-overlapping windows. We computed the genome-wide observed homozygosity (H_o_) and expected homozygosity (H_e_) at each SNP site using the ‘--hardy’ parameter in PLINK (v1.9) [33]. The computation of LD decay with physical distance between SNPs was performed using the PopLDdecay software (v3.41) [39] with the parameters ‘-MaxDist 500 -MAF 0.05’. We used GCTA software (v1.92) [35] with the parameter ‘--make-grm’ to compute a genomic relationship matrix (GRM) to obtain genomic relatedness among LSS individuals.

Following recent guidelines, SNP pruning was not applied before ROH detection [40]. The detection of ROHs in LSS and other breeds was carried out using PLINK (v1.9) [33] with the ‘--homozyg group’ command. The parameters used to detect ROH were as follows: ‘--homozyg-snp 10 --homozyg-kb 100 --homozyg-density 10 --homozyg-gap 100 --homozyg-window-snp 50 --homozyg-window-het 1 --homozyg-window-missing 5 --homozygwindow-threshold 0.05′, as described in the previous study using WGS data. Because ROHs with different lengths provide information on inbreeding at different past generations, the identified ROHs were divided into four length categories: 0.1–0.2 Mb, 0.2–0.5 Mb, 0.5–1 Mb, and >1 Mb.

### 2.5. Estimation of Genomic Inbreeding Coefficients

In brief, five methodologies were used to estimate genomic inbreeding coefficients in this study. PLINK(v1.9) [33] was used to calculate the excess of homozygosity inbreeding coefficient (*F*_HOM_), which is commonly used in livestock genetics. The *F*_VR1_ was estimated using the ‘--grm agrm --fmt 0’ command to obtain the diagonal elements of the GRM in GMAT software (v1.0) [41]. To calculate *F*_VR2_ and *F*_UNI_, we employed GCTA (v1.92) [35] with the ‘--ibc’ command. The advantage of the ROH inbreeding estimate lies in its capability to differentiate between recent and ancient inbreeding [42]. In LSS, we calculated the ROH-based inbreeding coefficient (*F*_ROH_), defined as the proportion of the autosomal genome (the total length of 2,472,477,635 bp in the ARS-UI_Ramb_v2.0) covered by the total ROH in each animal genome. Using the previously defined four ROH length categories [43], we further calculated *F*_ROH 0.1–0.2 Mb_, *F*_ROH 0.2–0.5 Mb_, *F*_ROH 0.5–1 Mb_, and *F*_ROH > 1 Mb_, which corresponds to 250–500 generations, 100–250 generations, 50–100 generations, and 50 generations ago, respectively.

### 2.6. Genome-Wide Identification of Selection Signals

To find the genetic loci affecting wool-related traits in LSS, we conducted genome-wide scans between LSS (a semi-fine-wool breed) and TIB (a coarser wool breed) using three complementary population statistics: cross-population extended haplotype homozygosity (XP-EHH) [44,45,46,47], *F*_ST_, and cross-population composite likelihood ratio (XP-CLR) [48]. All genomic regions were scanned using 10-kb non-overlapping sliding windows across the genomes of the BT and TIB populations. The 10-kb non-overlapping windows with the top 1% of the average value were identified as *F*_ST_, XP-EHH, and XP-CLR selection signals. We used Beagle software (v5.3) [49] to phase and impute SNPs. XP-EHH values for each SNP were calculated between BT and TIB populations using the selscan software (v2.0.0) [44]. The sliding window analysis of XP-EHH was ultimately performe with a 10-kb non-overlapping window. XP-CLR scores were calculated within windows using a maximum of 100 SNPs with the parameters (--phased --ld 0.95 --size 10,000 --step 10,000). The Manhattan diagram of the selection signals was created using the CMplot package in R language [50]. Additionally, genome-wide *F*_ST_ between BT and TIB animals was determined using VCFtools (v0.1.16) with the command ‘--fst-window-size 10,000’. Overlapping windows across the three complementary population statistics were considered as the ultimate candidate windows. After genome annotations, genes overlapping within these regions were identified as putative candidate genes. Functional enrichment analyses of these genes were conducted using the R package clusterProfiler (v 4.6.2) [51]. Enriched gene ontology (GO) terms with a *p* value < 0.05 were considered as significant.

## 3. Results

### 3.1. Abundant Genomic Variants in LSS

We obtained an average sequencing depth of 14.03× per individual in LSS, ranging from 11.36× to 24.83× (Supplementary Materials Table S1). A total of 21,017,024 SNPs (20,828,448 biallelic and 188,576 multiallelic) and 1,848,356 indels were detected across the autosomal genome. The functional annotation analysis revealed that genome-wide biallelic SNPs in LSS were mainly located in intronic regions (62.94%) or intergenic regions (25.12%), with only 1.15% of biallelic SNPs found in exon regions. Across the autosomal genomes of 139 sheep from six domestic sheep breeds and a wild sheep species (i.e., AMU), a total of 26,249,552 biallelic SNPs were identified, and the annotation results were similar to those observed in LSS (Supplementary Materials Table S2).

### 3.2. LSS Was Genetically Close to LST and RMN Sheep

**Figure 1:**
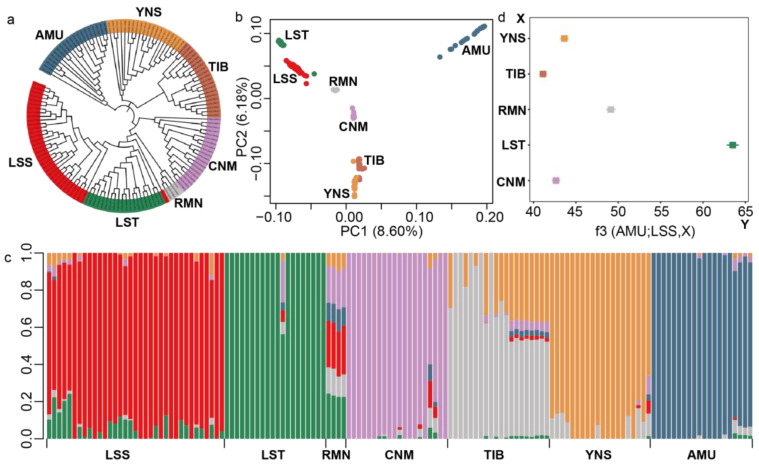
Population structure and genetic relationships among 6 domestic sheep breeds (*n* = 119) and 20 Asiatic mouflons (AMU). (**a**) Neighbor-joining (NJ) phylogenetic tree illustrating relationships among the six domestic sheep breeds (LSS: Liangshan semi-fine-wool sheep, LST: Border Leicester, RMN: Romney, CNM: Chinese Merino sheep, TIB: Tibetan sheep, and YNS: Yunnan sheep) and wild sheep. (**b**) Principal component analysis of all 139 sampled sheep. (**c**) Model-based clustering of sheep breeds using ADMIXTURE with K = 6. (**d**) The outgroup-f3 analysis of LSS with LST, RMN, CNM, TIB, and YNS, with AMU as an outgroup.

A NJ phylogenetic tree, constructed based on independent biallelic SNPs (LD, r^2^ > 0.2) showed that the LSS, LST, and RMN breeds grouped closely, where all animals from each of the seven sheep breeds formed distinct clusters (Figure 1a). In the PCA analysis, PC1 (first principal component) accounted for 8.60% of the total genetic variation, distinguishing between wild and domestic sheep, while PC2 (second principal component) represented 6.18% of the total genetic variation. It indicated the close clustering of LSS with LST and RMN which are both non-Chinese breeds (Figure 1b). LSS exhibited less genetic distance from the CNM breed than from TIB and YNS. The admixture analysis indicated that K = 6 (cross-validation error = 0.502, Supplementary Materials Figure S1) was the most likely number of genetically distinct breeds for the 139 samples. In this scenario, LSS exhibited a shared ancestral affinity with LST and RMN to some extent.

At the population level, the outgroup-f3 statistic (Figure 1d) revealed that LSS was genetically similar to LST and RMN. Furthermore, LSS showed lower genetic divergences (average weighted *F*_ST_ range from 0.086 to 0.087) with LST and RMN, compared to other domestic breeds.

### 3.3. Population Genomic Parameters and the Genome-Wide Pattern of ROH in LSS

**Figure 2:**
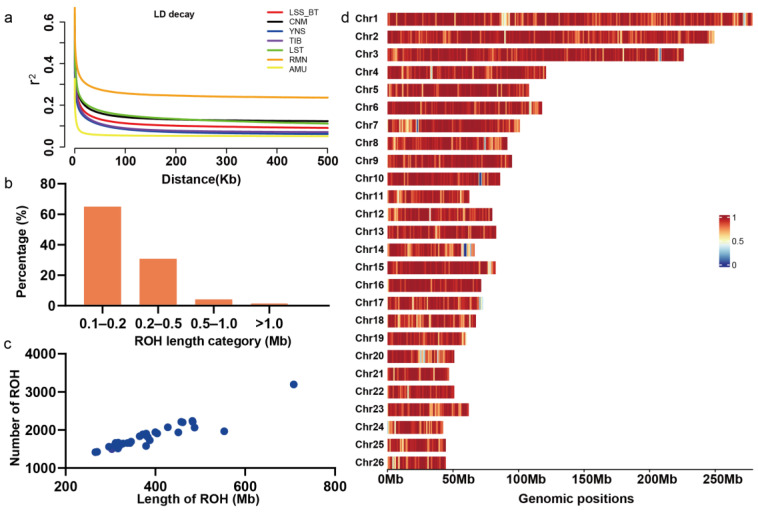
Summary of linkage disequilibrium (LD) and runs of homozygosity (ROH) detected in the sheep autosomal genome. (**a**) The genome-wide average LD decay estimated from each breed. (**b**) The proportions of the ROH total number with different length classes in LSS. (**c**) The summary of the total ROH number and length in the genome of each individual in LSS. (**d**) The distribution of the total ROH number and length across autosomes in LSS.

Since the WGS data for 35 LSS sheep were newly generated, we mainly focused on investigating the genetic diversity within this breed. Based on a genetic relationship matrix (GRM) analysis (Supplementary Materials Table S3), we found that most of the LSS samples were unrelated (SNP-derived genetic relatedness < 0.05). The total average observed heterozygosity (H_o_) and expected heterozygosity (H_e_) were 0.330 and 0.334, respectively, across all biallelic SNP sites. In the non-overlapping 10 kb window analysis, the genome-wide mean π value was 2.84 × 10^−3^, with a median π value of 2.60 × 10^−3^. The LD analysis (Figure 2a) showed that LSS exhibited the highest correlation (r^2^ = 0.580) with a 10-bp physical distance between SNPs. As the r^2^ value decreased to half of its maximum, the physical distance between SNPs within the LSS breed was approximately 2.5 kb, which was consistent with results observed in other sheep breeds (YNS, AMU, LST, TIB, and CNM), with distances ranging from 0.9 to 4.3 kb. Notably, RMN sheep exhibited a larger distance of 11.1 kb. The LD decay rate in LSS was faster than those in the CNM, LST, and RMN breeds but slower compared to the AMU, YNS, and TIB breeds.

In total, 63,588 ROHs were identified in the autosomal genomes of 35 LSS sheep. The descriptive statistics of ROH numbers and lengths by classes are detailed in Table 1. The total length of ROHs in LSS was composed mostly of a high count of shorter segments (ROH _0.1–0.2 Mb_), accounting for approximately 64.91% of all detected ROH but contributing to 42.75% of the cumulative ROH length. The distribution of ROH lengths approximated an L-shaped pattern in LSS (Figure 2b). The total ROH length per LSS individuals ranged from 266.15 Mb to 707.85 Mb (mean = 379.53 Mb, 15.35% of the autosomal genome), and this was positively correlated with the total ROH number in LSS individuals (Pearson’s *r* = 0.937, *p* < 2.2 × 2.2^−16^) (Figure 2c). The linear regression analysis demonstrated a significant increase in the number of ROH on chromosomes of greater length (Pearson correlation coefficient *r* = 0.825, *p* < 2.2 × 2.2^−16^) in LSS (Figure 2d). This trend of a positive linear relationship between chromosome length and the total number or length of ROHs was also observed in each chromosome within the LSS breed. A significant positive linear relationship between chromosome length and the total number of ROH or the total ROH length for each chromosome was also observed in each of the other six sheep breeds (Supplementary Material Table S4).

### 3.4. LSS Showed Low or Moderate Inbreeding Coefficients

**Figure 3:**
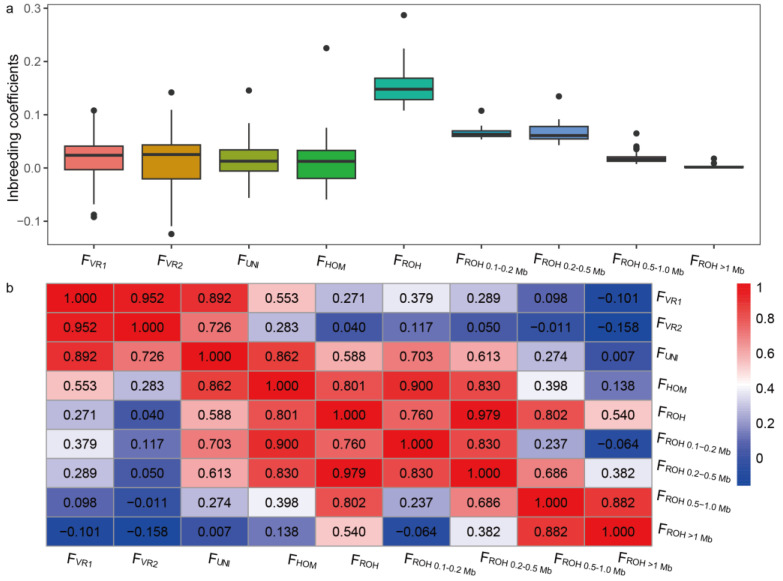
Genomic inbreeding coefficients in LSS were estimated using five different methods. (**a**) Boxplot displaying estimated genomic inbreeding coefficients in LSS. (**b**) Heatmap showing Pearson’s correlations among genomic inbreeding coefficients in LSS based on five methods.

The *F*_ROH_ per individual ranged from 0.108 to 0.287 in LSS sheep, with a median value of 0.148 (Figure 3a). An in-depth analysis revealed that the total genomic inbreeding coefficient levels mainly resulted from the inbreeding that occurred 250–500 (*F*_ROH 0.1–0.2 Mb_) and 100–250 (*F*_ROH 0.2–0.5 Mb_) generations ago at the population level. Compared to the *F*_ROH_, the results based on four other methods (*F*_VR1_, *F*_VR2_, *F*_UNI_, and *F*_HOM_) showed that the LSS breed has lower genomic inbreeding coefficients, with average values ranging from 0.014 to 0.016. 

As shown in Figure 3b, the strongest correlation (*r* = 0.952) was observed in *F*_VR1_ vs. *F*_VR2_, followed by *F*_VR1_ vs. *F*_UNI_, *F*_UNI_ vs. *F*_HOM_, and *F*_HOM_ vs. *F*_ROH_. The remaining comparisons displayed moderate to weak correlations, with the lowest correlation found between *F*_VR2_ and *F*_ROH_ (*r* = 0.04). Among the inbreeding values calculated from different ROH length subclasses, the highest correlation was found in *F*_ROH_ vs. *F*_ROH 0.2–0.5 Mb_ (0.979), followed by *F*_ROH_ vs. *F*_ROH 0.5–1.0 Mb_ (0.802).

### 3.5. Selective Signatures Associated with Wool Traits in LSS

**Figure 4:**
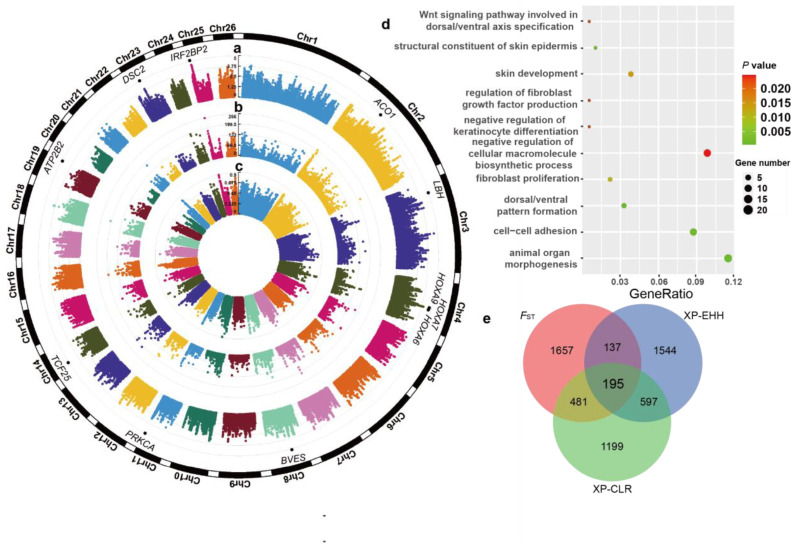
Genome-wide selection signals of LSS. Manhattan plot of genome-wide cross-population extended haplotype homozygosity (XP-EHH) (**a**), cross-population composite likelihood ratio (XP-CLR) (**b**), and *F*_ST_ (**c**) across all 26 autosomes between LSS and TIB sheep. (**d**) The enriched gene ontology (GO) biological processes for the genes overlapping with XP-EHH. (**e**) Venn diagram for the selected genes identified via three approaches.

Genome-wide XP-EHH statistics (Figure 4a) were calculated between LSS (semi-fine-wool) and TIB (coarse wool) to detect the positive selection signals in LSS. We detected 2473 putative outlier windows with XP-EHH larger than 2.464, overlapping with 637 annotated genes, including *IRF2BP2*, *ALOX5*, and *BVES*. The annotated genes were significantly enriched (*p* < 0.05) in 575 GO pathways (Supplementary Materials Table S5). Among these biological processes were fiber and wool development, such as the WNT signaling pathway involved in dorsal/ventral axis specification (*SFRP1*), cell–cell adhesion (16 genes, e.g., *ALOX5*, *BVES*, and *MAPK7*), fibroblast proliferation (4 genes, e.g., *IFI30*, *DACH1*, and *SFRP1*), and negative regulation of cellular macromolecule biosynthetic process (18 genes, e.g., *IRF2BP2*, *HOXA7*, *TCF25*, and *WNT11*) (Figure 4d). The selective sweep region with the highest genome-wide XP-EHH value (4.58) spanned 6,780,001–6,790,000 bp. The region harbored two genes on Chromosome 25 (i.e., *IRF2BP2* and *LOC101111733*).

Based on genome-wide XP-CLR and *F*_ST_ values (Figure 4b,c and Supplementary Materials Table S6), we identified 2472 and 2470 outlier windows exceeding the top 1% of windows (i.e., XP-CLR > 37.454 and *F*_ST_ > 0.500). All 195 candidate regions harboring 74 genes (e.g., *IRF2BP2*, *BVES*, *ALOX5*, and *HOXA7*) were ultimately determined as plausible selection signals in LSS (Figure 4e and Supplementary Materials Table S7). These candidate regions also overlapped with known quantitative trait loci (QTLs) related to wool traits in the Animal QTL database (https://www.animalgenome.org/, accessed on October 24, 2023), such as wool staple length (QTL: 14016), wool fiber trait (QTL: 14019), and wool fiber diameter (QTL: 14018). The 74 genes under positive selection exhibited significant enrichment in 150 GO biological processes (*p* < 0.05). In the significantly enriched GO biological processes, the top four were cAMP binding (two genes, *BVES* and *POPDC3*), cyclic nucleotide binding (two genes, *BVES* and *POPDC3*), negative regulation of response to endoplasmic reticulum stress (two genes, *ALOX5* and *USP25*), and negative regulation of transcription by RNA polymerase II (five genes, e.g., *IRF2BP2*, *HOXA7*, and *TCF25*).

**Figure 5:**
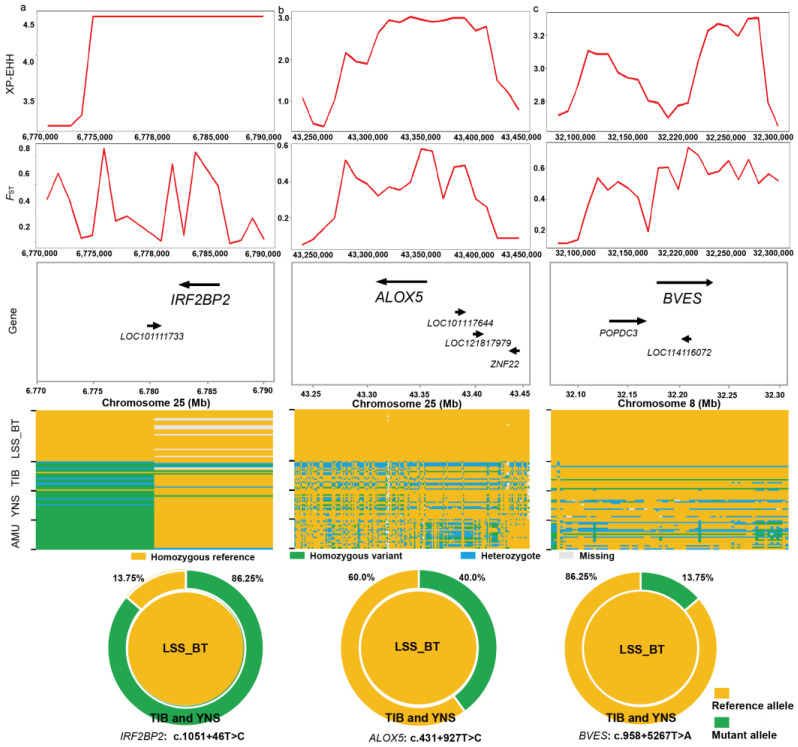
Summary of three examples of selective sweep regions. Three selection signals encompass *IRF2BP2* on Chromosome 25 (**a**), *ALOX5* on Chromosome 25 (**b**), and *BVES* on Chromosome 8 (**c**), respectively. These putative sweep regions were further validated by XP-EHH and *F*_ST_ values. Annotations of genes within the sweep region and SNPs fixed for reference alleles in LSS were provided. The distribution of allele frequencies at mutation sites in the three genes is shown at the bottom.

According to their biological functions and the distributions of SNPs within the corresponding regions, several genes were likely relevant to wool traits in LSS. For example, *IRF2BP2* (Chr25: 6,782,529–6,785,991 bp) was supported by high values of *F*_ST_ (average windowed *F*_ST_ = 0.534), in addition to the highest XP-EHH value (Figure 5a). There were four biallelic SNPs within the *IRF2BP2* gene, among which two SNPs (c.1052−253C > T and c.1051 + 46T > C, Chr25: 6,784,190 bp) in intron 1 were fixed for the reference alleles in LSS. By contrast, the reference allele frequency of the c.1051 + 46T > C was 13.75% in TIB and YNS sheep combined (22.5% in TIB and 5% in YNS sheep). The contingency table analysis revealed significant differences in genotypic frequency at this site between LSS and TIB/YNS sheep (Fisher’s exact test, *p* < 2.2 × 10^−16^). Furthermore, all AMU samples were homozygotes with the mutant allele at the c.1051 + 46T > C site, indicating the reference allele was a derived allele after domestication. 

As shown in Figure 5b, high XP-EHH (average windowed XP-EHH = 2.956) and *F*_ST_ values (average windowed *F*_ST_ = 0.454) revealed that the *ALOX5* gene (Chr25: 43,313,509–43,360,877 bp) was under strong selection in LSS. There were 527 biallelic SNPs within *ALOX5*, among which 188 SNPs were fixed for the reference alleles in LSS (Supplementary Materials Table S8). Particularly, the mutant allele frequency at the c.431 + 927T > C site (Chr25: 43,351,186 bp) was as high as 40% in TIB and YNS sheep combined (47.5% in TIB and 32.5% in YNS sheep). The contingency table analyses also revealed that the genotypic frequency at this site was significantly different between BT and TIB/YNS sheep (Fisher’s exact test, *p* = 1.2 × 10^−8^).

In addition, *BVES* (Chromosome 8: 32,174,087–32,228,333 bp) fell in a selective sweep that contained some windows with extremely high *F*_ST_ values (*F*_ST_ = 0.734) (Figure 5c). The reference alleles at 79 SNP sites within *BVES* reached fixation in LSS (Figure 5c and Supplementary Materials Table S8). Notably, there was a variant (c.958 + 5267T > A, Chr8: 32,209,881 bp) in intron 7 with a modifier functional impact. The reference allele frequency at the c.958 + 5267T > A was 13.75% in TIB and YNS sheep combined (17.5% in TIB and 10% in YNS sheep). A significant difference was found in the genotypic frequency of this site between LSS and TIB/YNS breeds (Fisher’s exact test, *p* = 1.4 × 10^−2^). 

## 4. Discussion

The LSS breed of sheep is a valuable genetic resource in China, because of the pro-duction of semi-fine-wool and its adaptability to the local environment. In this study, we employed high-density single-nucleotide polymorphisms (SNPs) obtained from whole-genome sequencing data to investigate the genetic relationships between LSS and five other domestic sheep breeds (i.e., TIB, YNS, CNM, LST, and RMN), as well as wild sheep (i.e., Asiatic mouflons). 

We employed multiple approaches with different genetic and statistical principles to analyze the genetic structure of the sampled sheep populations. For example, both phylogenetic and population structure analyses are used to cluster samples [28], while PCA analysis is used to study population structure by reducing the dimensionality of large-scale genetic data [29,43,52,53]. Although many individuals within each of the seven populations were determined to be closely related using the King software (v2.3.2) [54], population structure was similar among the sampled sheep with or without these closely related individuals. Compared to other domestic sheep populations (such as YNS), the results indicate that LSS generally showed closer genetic relationships with LST and RMN based on a variety of population genetic analyses, which is consistent with the genetic improvement history of LSS. Although CNM was introduced as a parental line in the breeding history of LSS, the relatively large genetic distance between LSS and CNM implied that CNM contributed a small fraction of the genetic composition of contemporary LSS. Recent studies have shown that the genome-wide average π value of the whole genome of domestic sheep falls within the range of 1.2 × 10^−3^ to 4.2 × 10^−3^ [55,56,57,58]. The whole-genome average π of LSS is 2.60 × 10^−3^, higher than that of LST but lower than AMU, in line with previous reports [58]. These results indicated that genetic variation in the genome of domestic sheep has been lost during the domestication process.

ROH is a stretch of homozygous genotypes in an individual that is thought to be mainly driven by accumulated inbreeding within a population [59]. Furthermore, several other factors may contribute to the presence of ROH in the genome, primarily including selection pressure, genetic drift, and population demographic events [60,61]. We examined the genome-wide ROH patterns in LSS by leveraging WGS data. It is known that ROH artifacts may arise due to suppressed recombination, substantial SNP gaps, and various other factors. Our analysis addressed ROH artifacts by focusing on ROH with a minimum length of 100 kb, effectively reducing artifacts caused by high LD between SNPs [62]. We also set a higher minimum SNP density to control ROH artifact reduction resulting from large SNP gaps, as exemplified in previous studies [40,62,63]. Therefore, we obtained high-quality ROH results in LSS. The number of ROHs observed in LSS was significantly higher than in many other sheep populations [64,65], potentially attributable to the detection of millions of SNPs. Among different ROHs, the ROH _0.1–0.2 Mb_ proportion was the highest, and the distribution of ROH lengths in the LSS genome closely followed an L-shaped distribution, consistent with results in sheep [66], goats [40,53], cattle [67], and pigs [68]. Detailed analyses indicate that the ROH patterns in LSS genomes primarily stem from historical inbreeding among individuals.

Compared to traditional pedigree-based coefficients, genomic inbreeding coefficients effectively capture the cumulative effects of inbreeding resulting from common ancestors across generations [69]. In this study, the four methodologies (i.e., *F*_VR1_, *F*_VR2_, *F*_UNI_, and *F*_HOM_) provided comparable inbreeding estimates, among which the first three were based on genomically realized relationship matrices among individuals. These methods, which are associated with genotype allele frequency and gamete union, significantly influence the inbreeding coefficient [35]. When defining inbreeding concepts, it is crucial to consider these unique attributes, as exemplified by the UNI method, which encompasses the definitions proposed by Wright [70]. The estimation of inbreeding coefficients in the majority of molecular data is primarily based on marker allele identity and generally does not directly differentiate between regions that are identical by descent and those that are identical by state. The advantage of the ROH-based inbreeding estimation, aside from its independence on allele frequency, is its capacity to distinguish between recent and ancient inbreeding. ROH-based inbreeding coefficients have found widespread application in the estimation of inbreeding and inbreeding depression in diverse livestock species, including sheep [59,71], goats [43,72], cattle [73,74,75], pigs [76,77,78], and poultry [79]. It is noteworthy that, in line with previous studies [75], a strong correlation between *F*_ROH_ and *F*_HOM_ was observed in LSS, as both metrics encompass whole-genome homozygosity. This highlights the varying levels of sensitivity in reflecting inbreeding across different periods among some inbreeding estimation methods. Regarding the previous *F*_ROH_ criteria, the majority of LSS demonstrate moderate values (0.1 < *F*_ROH_ < 0.2), aligning with those observed in Cyprus Fat-Tailed sheep and falling below the levels found in AMU sheep [61]. Taken together, it is necessary to prevent high inbreeding to avoid the potential loss of genomic diversity in LSS.

Here, we integrated three complementary population statistics to accurately search for plausible signatures of selection in LSS. The genes under selection (e.g., *IRF2BP2*, *BVES*, and *ALOX5*) were significantly enriched in the GO terms related to fiber and wool development (e.g., cell–cell adhesion, fibroblast proliferation, WNT signaling pathway involved in dorsal/ventral axis specification, and negative regulation of cellular macromolecule biosynthetic process). In mice, hair follicle formation is initiated by a dermal signal, where the activation of WNT signaling is crucial and precedes the localized expression of regulatory genes, leading to the initiation of hair follicle placode formation [80]. Dermal fibroblasts are essential for maintaining the structural integrity of the skin and supporting the development of hair follicles [81]. Using inducible and knockout mouse models, a previous study suggested that cell adhesion and orientation within hair germ contribute significantly to the precise determination of cell fate and the process of hair morphogenesis through the miR-200 family [82].

In this study, we found a strong selection signal associated with the *IRF2BP2* gene known as a major locus and a strong selection signal affecting fleece fibers in previous studies [18,19,83,84]. Specifically, differences between hairy and woolly fleece have previously been linked to the presence of an antisense *EIF2S2* retrogene insertion in the 3′-UTR of *IRF2BP2*, using a predominant French population dataset [19]. Among the detected SNPs in *IRF2BP2*, the genotype frequency at the mutation site (c.1051 + 46T > C, Chr25: 6,784,190 bp) showed maximum differences between BT and TIB and YNS sheep. However, selection signals in multiple sheep populations and related results strongly suggested that a novel mutation (Chr25: T7,068,586C) located in the 3′ UTR of *IRF2BP2* may serve as a potential causal variant influencing wool fiber diameter [18]. After accounting for the influence of reference genome disparities (Oar_rambouillet_v1.0 reference genome), these findings suggest that distinctive mutations may be attributed to breed-specific variations, indicating that the identification of the genuine causal variant sites for fleece fiber diameter would require further molecular studies and large samples. Additionally, all wild sheep are homozygous at this site (Chr25: 6,784,190 bp), indicating that the site arose in sheep post-domestication. In summary, marker-assisted selection based on the SNPs within *IRF2BP2* may improve the accuracy of selection for fleece fiber in LSS. 

This study also revealed strong selection pressure on *ALOX5* and *BVES* genes within the LSS population. Previous work has shown that *ALOX5* and *BVES* are associated with skin and hair follicle development [18,85]. *BVES*, identified as a transmembrane protein, is closely linked to blood vessels and epicardial tissue. It is commonly expressed in cell adhesions within the skin, in developmental embryos, and in mature adults [18]. Compared with other populations, all LSS sheep carried homozygous genotypes at 79 SNP sites in *BVES*, indicating that LSS is subjected to relatively intensive positive selection. It is well known that the stratum corneum of the skin is a surface layer composed of keratinocytes and serves as a barrier to the external environment [86]. Our enrichment analysis suggested that the *BVES* gene is mainly associated with cell tight-junction signaling pathways, such as cell–cell adhesion, cell-substrate adhesion, and anchoring junction. Therefore, this gene has the potential to influence the thickening or damage of the stratum corneum by regulating these pathways, leading to the differential development of hair follicles [87,88]. In the context of processes affecting hair follicles, arachidonic acid metabolism, a pathway encompassing the *ALOX5* gene, is particularly noteworthy [85]. The differential expression of the *ALOX5* gene in curly versus straight hair reflects its potential involvement in hair morphology, homeostasis, development, and shaping [85]. The c.431 + 927T > C mutation within the *ALOX5* gene exhibits a high allelic frequency of 40% in Tibetan and Yunnan sheep, suggesting a potential influence on wool fineness through the arachidonic acid metabolism pathway.

## 5. Conclusions

Among the six domestic sheep populations studied, the LSS, LST, and RMN breeds were closely related, while LSS showed significant genetic difference from TIB and YNS. Notably, the LD decay in LSS occurred at a faster rate compared to several other sheep breeds like AMU, YNS, and TIB. In terms of the inbreeding values, LSS typically exhibited moderate levels of inbreeding. Additionally, specific genes, such as *IRF2BP2*, were pinpointed in LSS, potentially representing the genetic foundation for adapting to local environmental conditions and potentially associated with wool traits.

## Figures and Tables

**Table 1:** Descriptive statistics of runs of homozygosity (ROH) number and length by ROH length class.

ROH Class	n ROH	Mean Length (kb)	Median Length (kb)	Standard Deviation	Genome Coverage (%)
0.1–0.2 Mb	41274	137.57	132.02	27.60	6.56
0.2–0.5 Mb	19544	293.39	273.17	76.36	6.62
0.5–1 Mb	2587	638.84	604.20	120.10	1.91
>1 Mb	183	1195.06	1134.59	200.11	0.25

## Data Availability

The data presented in this study are available on request from the corresponding author. The data are not publicly available because other results of this dataset has not been released yet.

## Supplementary Materials

The following supporting information can be downloaded at: https://www.mdpi.com/article/10.3390/ani14030444/s1, Figure S1: Model-based clustering of sheep breeds using ADMIXTURE with K values ranging from two to seven; Table S1: Summary of the short-read sequencing data and mapping statistics for Liangshan semi-fine-wool sheep breed; Table S2: Distribution of the identified biallelic SNPs in 139 sheep genomes within various genomic regions; Table S3: A genomic relationship matrix in LSS sheep; Table S4: The linear relationship between the ROH (ROH length and number) on each chromosome and the chromosome length; Table S5: The enriched GO biological processes for the genes with XP-EHH; Table S6: Genome-wide detection of XP-EHH, XP-CLR, and *F*_ST_ selection signals in LSS sheep; Table S7: Summary of the selected genes identified via three approaches; Table S8: Summary of SNPs within three genes fixed for reference alleles in Liangshan semi-fine-wool sheep.
