# Supplementary material for: Whole-Genome Resequencing Reveals Genetic Diversity and Wool Trait-Related Genes in Liangshan Semi-Fine-Wool Sheep
Source: Animals (Basel). 2024 Jan 29;14(3):444. doi: 10.3390/ani14030444 (PMC10854784; doi:10.3390/ani14030444)
Supplement: Supplementary file 1 [file animals-14-00444-s001.zip › Table S1.docx]

Table S1 Summary of the short-read sequencing data and mapping statistics for Liangshan Semi-fine wool sheep breed

| Sample | Mapping ratio (%) | Genome coverage (%) | Sequencing depth (×) |
| --- | --- | --- | --- |
| LSS01 | 99.87 | 99.16 | 15.74 |
| LSS02 | 99.91 | 99.00 | 11.36 |
| LSS03 | 99.91 | 99.06 | 12.47 |
| LSS04 | 99.91 | 99.02 | 12.01 |
| LSS05 | 99.92 | 99.42 | 13.58 |
| LSS06 | 99.90 | 99.03 | 12.54 |
| LSS07 | 99.92 | 99.56 | 21.92 |
| LSS08 | 99.94 | 99.32 | 12.29 |
| LSS09 | 99.89 | 99.16 | 16.80 |
| LSS10 | 99.94 | 99.34 | 12.23 |
| LSS11 | 99.90 | 99.47 | 15.54 |
| LSS12 | 99.90 | 99.10 | 13.74 |
| LSS13 | 99.94 | 99.08 | 13.37 |
| LSS14 | 99.91 | 99.08 | 13.37 |
| LSS15 | 99.94 | 99.06 | 13.49 |
| LSS16 | 99.87 | 99.06 | 12.70 |
| LSS17 | 99.85 | 99.08 | 12.86 |
| LSS18 | 99.90 | 99.05 | 12.20 |
| LSS19 | 99.92 | 99.06 | 12.47 |
| LSS20 | 99.91 | 99.03 | 12.67 |
| LSS21 | 99.93 | 99.06 | 12.60 |
| LSS22 | 99.92 | 99.32 | 12.41 |
| LSS23 | 99.91 | 99.35 | 12.35 |
| LSS24 | 99.90 | 99.35 | 12.29 |
| LSS25 | 99.91 | 99.36 | 12.52 |
| LSS26 | 99.87 | 99.36 | 12.11 |
| LSS27 | 99.93 | 99.34 | 11.48 |
| LSS28 | 99.88 | 99.32 | 11.54 |
| LSS29 | 99.92 | 99.42 | 13.58 |
| LSS30 | 99.91 | 99.07 | 12.65 |
| LSS31 | 99.84 | 99.55 | 24.83 |
| LSS32 | 94.90 | 99.20 | 21.63 |
| LSS33 | 99.76 | 99.15 | 15.70 |
| LSS34 | 99.85 | 99.14 | 16.38 |
| LSS35 | 90.17 | 99.47 | 15.66 |
