# Supplementary figures and images for: Whole-Genome Resequencing Reveals Genetic Diversity and Wool Trait-Related Genes in Liangshan Semi-Fine-Wool Sheep
Source: Animals (Basel). 2024 Jan 29;14(3):444. doi: 10.3390/ani14030444 (PMC10854784; doi:10.3390/ani14030444)

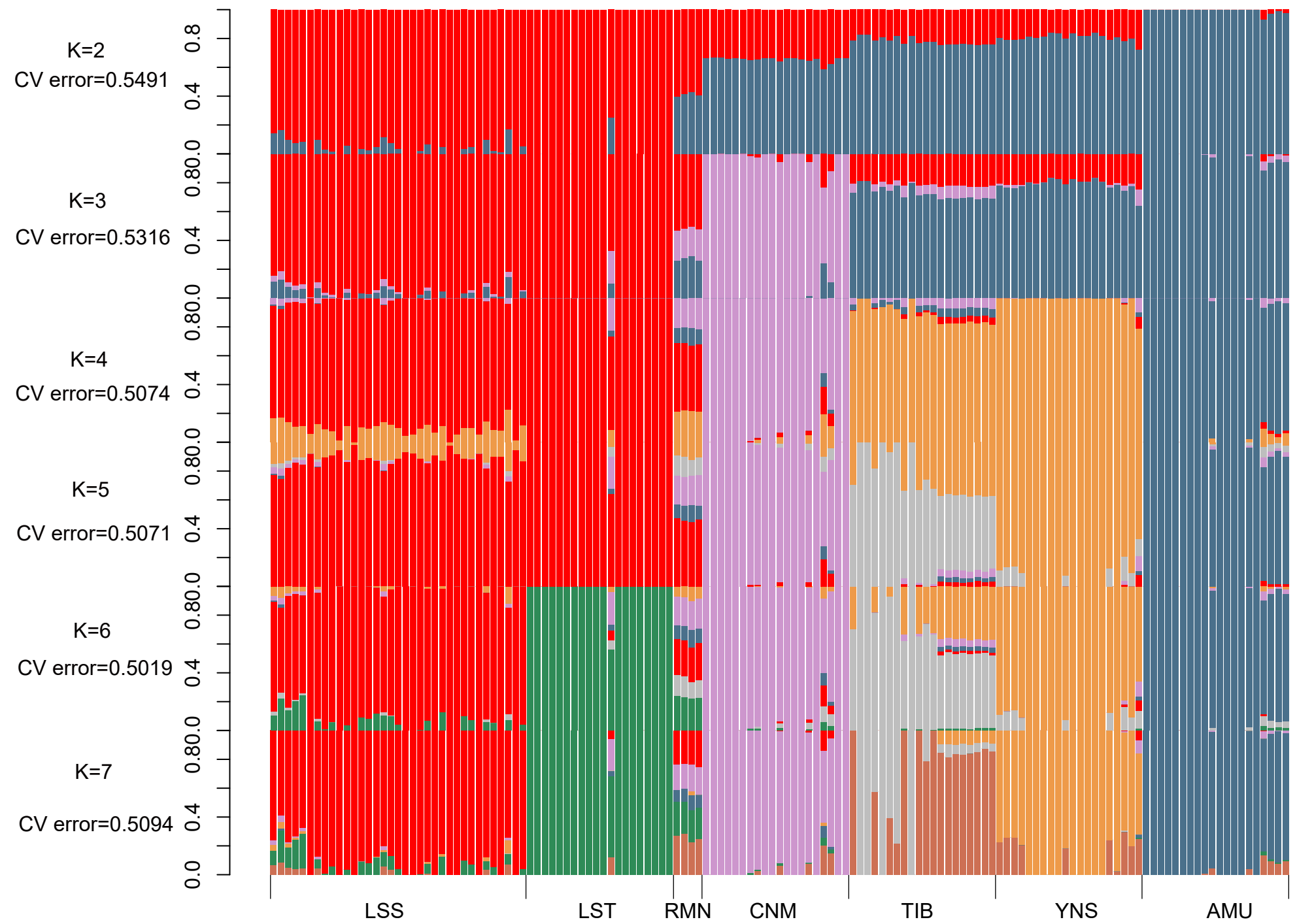

Supplement: Supplementary file 1 [file animals-14-00444-s001.zip › Figure S1.pdf]
